# Effects of Linguistic Labels on Visual Attention in Children and Young Adults

**DOI:** 10.3389/fpsyg.2018.00358

**Published:** 2018-03-21

**Authors:** Wesley R. Barnhart, Samuel Rivera, Christopher W. Robinson

**Affiliations:** ^1^ID/ASD Research Group, Nisonger Center, University Center for Excellence in Developmental Disabilities, The Ohio State University, Columbus, OH, United States; ^2^Department of Psychology, The Ohio State University, Columbus, OH, United States; ^3^Department of Psychology, The Ohio State University at Newark, Newark, OH, United States

**Keywords:** linguistic labels, visual attention, eye tracking, development

## Abstract

Effects of linguistic labels on learning outcomes are well-established; however, developmental research examining possible mechanisms underlying these effects have provided mixed results. We used a novel paradigm where 8-year-olds and adults were simultaneously trained on three sparse categories (categories with many irrelevant or unique features and a single rule defining feature). Category members were either associated with the same label, different labels, or no labels (silent baseline). Similar to infant paradigms, participants passively viewed individual exemplars and we examined fixations to category relevant features across training. While it is well established that adults can optimize their attention in forced-choice categorization tasks without linguistic input, the present findings provide support for label induced attention optimization: simply hearing the same label associated with different exemplars was associated with increased attention to category relevant features over time, and participants continued to focus on these features on a subsequent recognition task. Participants also viewed images longer and made more fixations when images were paired with unique labels. These findings provide support for the claim that labels may facilitate categorization by directing attention to category relevant features.

## Introduction

Through communication, we are able to gain valuable experiences and knowledge of our environment; thus, it is not surprising that researchers and philosophers have long speculated about possible links between language and thought, with language possibly playing a causal role in the formation of percepts and concepts (Whorf, 1956; Gentner and Goldin-Meadow, 2003; Gleitman and Papafragou, 2005; Lupyan, 2012). It is well-established that adults understand the symbolic nature of language and treat linguistic labels differently than other features (Yamauchi and Markman, 2000), and exposure to labels appears to facilitate categorization—the ability to treat discriminable exemplars as equivalent (Lupyan et al., 2007). Developmental research shows that these effects have an early onset with spoken words and speech sounds, but not other types of auditory information, influencing categorization, individuation, and induction in 3- to 21-month-old infants (Balaban and Waxman, 1997; Welder and Graham, 2001; Xu, 2002; Fulkerson and Haaf, 2003; Plunkett et al., 2008; Ferry et al., 2010, see also Robinson et al., 2012 for a review). While effects of labels on various cognitive tasks are well-established, underlying mechanisms, especially early in development, are hotly debated.

Developmental studies examining the *outcome* of learning show that exposure to linguistic labels affect the categories that infants learn. For example, when 3- to 13-month-old infants hear the same label associated with multiple exemplars they often form one category, whereas when exemplars are associated with different labels infants often individuate items or form multiple categories (Waxman and Markow, 1995; Balaban and Waxman, 1997; Xu, 2002; Plunkett et al., 2008; Ferry et al., 2010; Althaus and Westermann, 2016; Havy and Waxman, 2016). To account for these findings, it has been proposed that hearing the same label paired with multiple exemplars may facilitate categorization by directing visual attention to common features (cf. Waxman, 2003; Lupyan, 2012), and given that unique labels facilitate individuation and learning of multiple categories, it is also possible that hearing unique labels associated with different exemplars may direct attention to individual features and away from commonalities.

The few developmental studies that have used an eye tracker to directly test the hypothesis that labeling facilitates category learning by highlighting category relevant features are mixed. For example, support for the hypothesis primarily comes from work by Althaus and colleagues (Althaus and Mareschal, 2014; Althaus and Plunkett, 2015a,b; Althaus and Westermann, 2016). Using variations of familiarization procedures, these studies demonstrate that by 10- to 12-months of age labels direct attention to commonalities, with infants in the label conditions often requiring less familiarization before looking at common features (Althaus and Mareschal, 2014). It is also important to note that these effects do not appear to generalize to other types of auditory cues such as nonlinguistic sounds (Althaus and Mareschal, 2014; Althaus and Westermann, 2016). Infants are also sensitive to the timing of the linguistic labels, with synchronous timing of auditory and visual information sometimes interfering with familiarization (Althaus and Plunkett, 2015b). Finally, there is some evidence that effects of labels on visual attention occur before the labels are presented (Althaus and Mareschal, 2014). More specifically, labels in Althaus and Mareschal (2014) were presented at approximately 1020 ms after visual stimulus onset; however, effects of labels on attention were found when examining the first 1000 ms of the trial (prior to the presentation of the count noun). In summary, while effects of labeling on visual attention appear to be fragile with small changes in timing affecting learning, facilitative effects, when found, appear to have lasting consequences on attention, with infants continuing to focus on common features on a subsequent testing phase even when no labels are presented (Althaus and Plunkett, 2015a).

At the same time, other studies have failed to find support for the hypothesis that labels highlight common features (Best et al., 2010, 2011; Deng and Sloutsky, 2015). For example, 6- to 8-month-old infants and 4-year-olds in Best et al. (2010, 2011) were familiarized (infants) or presented with a series of stimuli on a categorization task (children), and images were either labeled or presented in silence. Half of the features on visual stimuli were unique and varied from exemplar to exemplar, whereas the other half were common (category members all shared the same features). Both infants and children accumulated more looking to the unique features, with no evidence that labels directed attention to the common, category relevant information. This finding is inconsistent with the claim that labels highlight commonalities; however, it is likely that infants’ and children’s attention was more bottom-up in nature and captured by the changing unique features (i.e., novelty preference). Thus, it is possible that the discrepancy in previous research stems from using variations of familiarization/novelty preference paradigms, which might not be optimal if the task is to focus on unchanging features.

Discrepancies across the studies might also stem from some multisensory conditions interfering with visual processing. For example, presenting linguistic input and visual stimuli at the same time can attenuate categorization in 12-month-olds (Althaus and Plunkett, 2015b) and facilitation effects of labels disappear when the presentation of speech is not contingent on 15-month-old infants’ looking (Roberts, 1995). Auditory dominance studies also show that the presence of an auditory stimulus, including spoken words, can interfere with visual processing early in 8- to 16-month-old infants and 4-year-olds (Lewkowicz, 1988a,b; Sloutsky and Napolitano, 2003; Robinson and Sloutsky, 2004, 2007b; Sloutsky and Robinson, 2008; Nava and Pavani, 2013). While many of these studies show that infants and 4-year-olds are less likely to discriminate two images when paired with sounds or words, other studies have shown that some of these effects also occur on higher-level tasks such as categorization and individuation (Robinson and Sloutsky, 2007a, 2008). For example, 8- and 12-month-old infants in Robinson and Sloutsky (2007a) were more likely to categorize visual stimuli when images were paired with linguistic labels than nonlinguistic sounds; however, both labels and sounds interfered with visual categorization compared to a silent condition. Given auditory dominance findings and infants’ and children’s sensitivities to the labeling contexts, it is also important to examine effects of linguistic labels on attention and categorization in older participants.

Adults treat linguistic labels differently than other features (Yamauchi and Markman, 2000). For example, in a classification task, adults were presented with a novel creature and they had to determine if the creature belonged to category 1 or category 2. In the induction task, adults were presented with a novel creature and a linguistic label and they had to make an inference about the appearance of a hidden feature. If linguistic labels are simply perceptual features, then participants should respond similarly on both tasks because they were presented with the same number of correlated features. However, adults relied almost exclusively on the linguistic labels in the induction task, suggesting that adults treated linguistic labels differently than other perceptual features.

Linguistic labels also appear to affect the outcome of category learning in adults (Lupyan et al., 2007). For example, in Lupyan et al. (2007), adults were familiarized to two novel categories. On each trial, they were presented with a novel creature and they had to make a forced-choice category judgment (i.e., move toward or away from creature). Participants were provided with feedback after each response, and in the label condition, they were also presented with a written or spoken name for the creature after they made their response. Adults were faster at learning the categories and were also more accurate in a subsequent testing phase when linguistic labels were perfectly correlated with category membership. However, visual fixations were not collected while learning the novel categories; thus, it is unclear if labels facilitated category learning by directing attention to category relevant features.

Selective attention and adjusting attentional weights to reduce error are important components in categorization models (see Rehder and Hoffman, 2005; Blair et al., 2009 for related discussions). Eye tracking research has demonstrated that adults are skilled in attention optimization and increase looking to category relevant features (Rehder and Hoffman, 2005; Blair et al., 2009); however, this research has not examined how labels affect attention optimization. For example, participants in Blair et al. (2009) were presented with novel stimuli (e.g., four different types of fictitious microorganisms) and they had to use two of the three features to correctly classify each microorganism. Feedback was provided after each trial. Initially, participants showed diffused patterns of attention to relevant and irrelevant features; however, after successfully categorizing the microorganisms, adults optimized their attention and selectively focussed on the category relevant features.

While previous developmental research testing the hypothesis that labels direct attention to commonalities often used basic or dense categories (Best et al., 2010, 2011), effects of labels appear to be important for learning of sparse categories (Perry and Lupyan, 2016), and it is possible that some of the discrepancies in previously reported research stems from using dense categories that can be learned without supervision. Dense categories typically have a high family resemblance and have many correlated features (e.g., birds typically have beaks, feathers, small bodies, chirp, etc.), sparse categories typically have many irrelevant or unique features with a small subset of features that correlate or define the category (e.g., stripes on a zebra), and these categories are more difficult to learn than dense categories, especially early in development (Kloos and Sloutsky, 2008; Sloutsky, 2010). Moreover, selective attention abilities correlate with categorization of sparse categories. In particular, adults’ performance on a flanker task requiring them to specify the direction of a central target while ignoring irrelevant distractors on either the right or left sides (measure of selective attention) was associated with how quickly participants verified the names of labels denoting sparse categories (Perry and Lupyan, 2016). On a related note, there is evidence with 9- to 15-month-old infants that exposure to linguistic input has a larger effect on learning of sparse or more general categories, whereas, dense/basic categories can often be learned without exposure to labels (Waxman and Markow, 1995; Fulkerson and Haaf, 2003). Thus, labeling and selective attention might be crucial on tasks that require focusing on a small subset of features/dimensions while simultaneously ignoring many irrelevant features/dimensions.

In summary, previous research examining effects of labels on categorization in adults typically focuses on learning rates and the outcome of learning (not on changes in visual attention during learning), and research examining attention optimization typically does not label the visual categories. In addition, most of the categorization research relies on an explicit categorization task where participants have to make category judgments and are often provided with feedback about category membership after each response. Thus, a gap exists in the literature in terms of if simply hearing labels for novel images can lead to label induced attention optimization and direct attention to category relevant features.

To fill this gap, the present study employed a novel procedure to examine fixations as 8-year-olds and adults were simultaneously familiarized to novel creatures from three different sparse categories. Given the discrepancies found in infant and child research testing effects of labels on attention (Best et al., 2010, 2011; Althaus and Mareschal, 2014; Althaus and Plunkett, 2015a,b; Deng and Sloutsky, 2015; Althaus and Westermann, 2016), auditory overshadowing effects found in infants and young children (Lewkowicz, 1988a,b; Sloutsky and Napolitano, 2003; Robinson and Sloutsky, 2004, 2007b; Sloutsky and Robinson, 2008; Nava and Pavani, 2013), the difficulty of the present task with the simultaneous presentation of three categories, and the fact that we trained participants on sparse categories which are difficult for young children (Kloos and Sloutsky, 2008; Sloutsky, 2010), we recruited 8-year-old children who should have better attentional control and should be less affected by simultaneously presented auditory information. Given that child participants were significantly older than children used in previous research, we were not expecting to find age differences in looking to category relevant features.

The use of sparse stimuli was important because attention optimization appears to be crucial for learning categories with high within-category variability and a single diagnostic feature (Perry and Lupyan, 2016, see also Waxman and Markow, 1995; Fulkerson and Haaf, 2003, for stronger effects of labeling on broad categories). Similar to infant familiarization procedures, participants were not told about category membership and they simply viewed the stimuli while hearing some of the names for the creatures. Note that this paradigm differs considerably from many adult categorization studies where participants are instructed to make category judgments about each stimulus, which is followed by corrective feedback. This absence of explicit instructions on category membership/feedback made it possible to examine the primary question of the present study: Does *simply hearing* labels for novel creatures result in label induced attention optimization? Category members were presented in silence (baseline), paired with a common label (same label paired with all members of the category), and paired with unique labels (different labels associated with members from the category). Based on proposed mechanisms underlying effects of labels on category learning (Waxman, 2003; Lupyan, 2012), it was hypothesized that common labels would help direct children’s and adults’ attention to category relevant features and unique labels would help participants direct attention to unique features (features that differentiate category members).

We also examined recognition of individual items at test, as opposed to testing categorization, to determine if effects of labels carry over to non-categorization tasks. For example, 4-year-olds and adults in Sloutsky et al. (2005) were trained on two items that were either associated with the same label or presented in silence. During test, participants had to determine if two images were identical or different. Even though no labels were presented at test, children’s discriminability of trained stimuli decreased if they heard labels during training, suggesting that some of the effects of labeling may have more general effects and carry over to non-categorization tasks. In contrast, there was no evidence that the labels presented during training attenuated adults’ subsequent discriminations. The present study expands on this research by examining patterns of visual fixations and discrimination accuracy on a subsequent object recognition task.

## Materials and Methods

### Participants

Fifteen 8-year-olds (5 Females, *M* = 8.80 years, *SE* = 2.48) and twenty-four adults (13 Females, *M* = *18.71* years, *SE* = 4.30) participated in the study. Children were recruited by word-of-mouth and young adults were recruited from a Midwestern university. Participants completed the study in a quiet testing room. After participants/legal guardians were informed about the nature of the present study, adults completed an IRB approved informed consent form. All children in the study verbally assented to participate and guardians also filled out an IRB approved parental consent form. All participants had normal hearing and vision (as reported by participants/guardians) and were debriefed after study completion. Recruitment and experimental procedures were carried out in accordance with guidelines and approval of the university’s Institutional Review Board. Six additional participants (two children and four adults) were tested but excluded due to poor eye tracking calibration.

### Stimuli and Eye Tracking Acquisition

**FIGURE 1:**
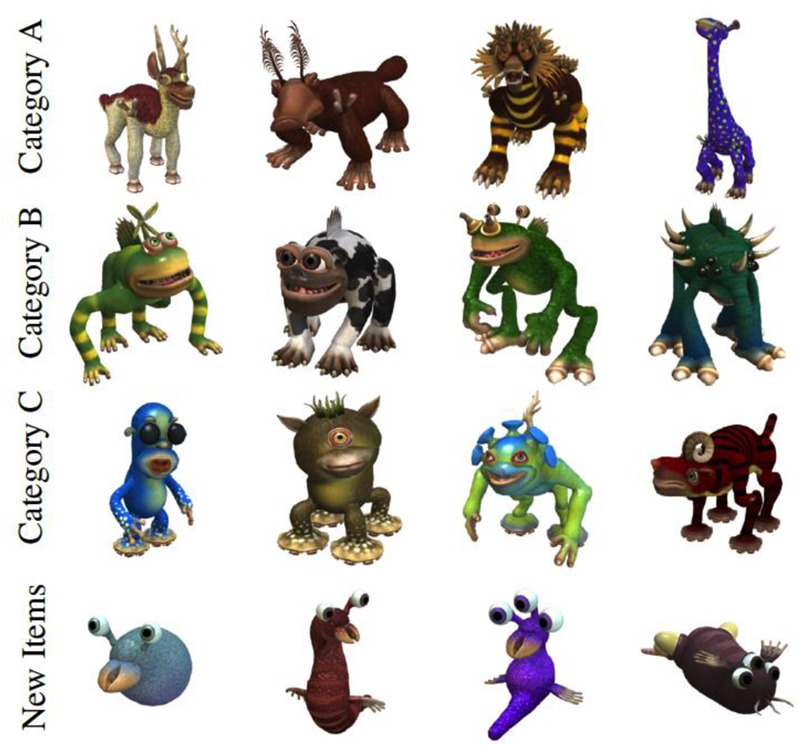
Example stimuli used in the experiment. Each category is defined by the presence of a particular feature: prongs on the shoulders for Category A, dorsal fin for Category B, and suction cup feet for Category C.

**FIGURE 2:**
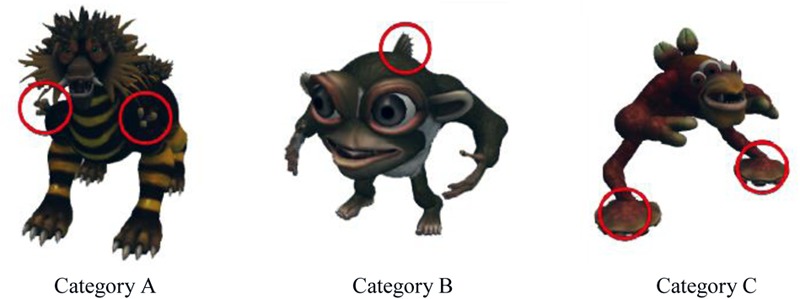
Examples of stimuli and Areas of Interest (AOI). Red circles denote AOIs around the category relevant features.

Verbal labels were pre-recorded sound clips of count nouns in child directed carrier phrases (e.g., “Look, here’s a dax”, “this is called a dax”, etc.) that were spoken by a female experimenter. Sound clips were recorded using a Yeti Pro microphone and edited using Audacity software so that each linguistic phrase was approximately one second in duration. Auditory stimuli were presented via Kensington KMW33137 headphones at approximately 65 dB and the carrier phrase shared the same onset as the visual stimulus. Visual stimuli were artificial creatures generated with the Spore creature creation software (Electronic Arts Inc., 2008), and participants determined how long each visual stimulus was presented (discussed more below). Creatures were from three different categories defined by the presence of a single deterministic feature (see **Figure 1** for examples of visual stimuli). All other features varied independently of the category and gave no information about category membership. Category A was defined by the presence of three small prongs on the front shoulders. Category B members had a dorsal fin. Category C creatures had suction-cup feet. On average, stimuli spanned approximately 18 by 13 degrees of visual angle and were presented on a 1920 × 1080 Benq XL2420-B monitor. The area of interest (AOI) for each creature was a fixed size circle (or circles) enclosing the deterministic feature(s). Some example AOIs are shown in **Figure 2**. Category A AOIs consisted of one or two 3-prong sections located on the upper half of the stimulus (depending on the orientation of the creature); Category B only had a single AOI which was a dorsal fin located on the upper half of the stimulus; and Category C AOIs consisted of two or four suction cup feet located on the lower half of the stimulus. Category type (A, B, or C) and Labeling (common, unique, no label) were counterbalanced across participants.

Gaze data were recorded during training and testing at 500 Hz by an Eyelink 1000 Plus Tower system (SR Research, ON, Canada). Fixation information was identified on-line during the experiment by using the default settings in the Eyelink Plus system, and then recorded for offline processing and analysis with custom MATLAB and Python software. The Eyelink system identifies fixations as periods when the gaze does not exceed saccadic motion thresholds. Saccades must exceed a visual angle velocity of 30 degrees per second or acceleration of 8000 degrees per second squared while having a displacement of at least 0.1 visual degrees. Adults were calibrated with a 9 point calibration by using the default Eyelink 1000 settings. To simplify calibration, children were calibrated using a 5-point calibration. Adults’ average calibration errors during training and test were 0.87 and 0.81 degrees, respectively, and children’s average calibration errors during training and test were 0.59 and 0.81 degrees, respectively.

### Procedure

**FIGURE 3:**
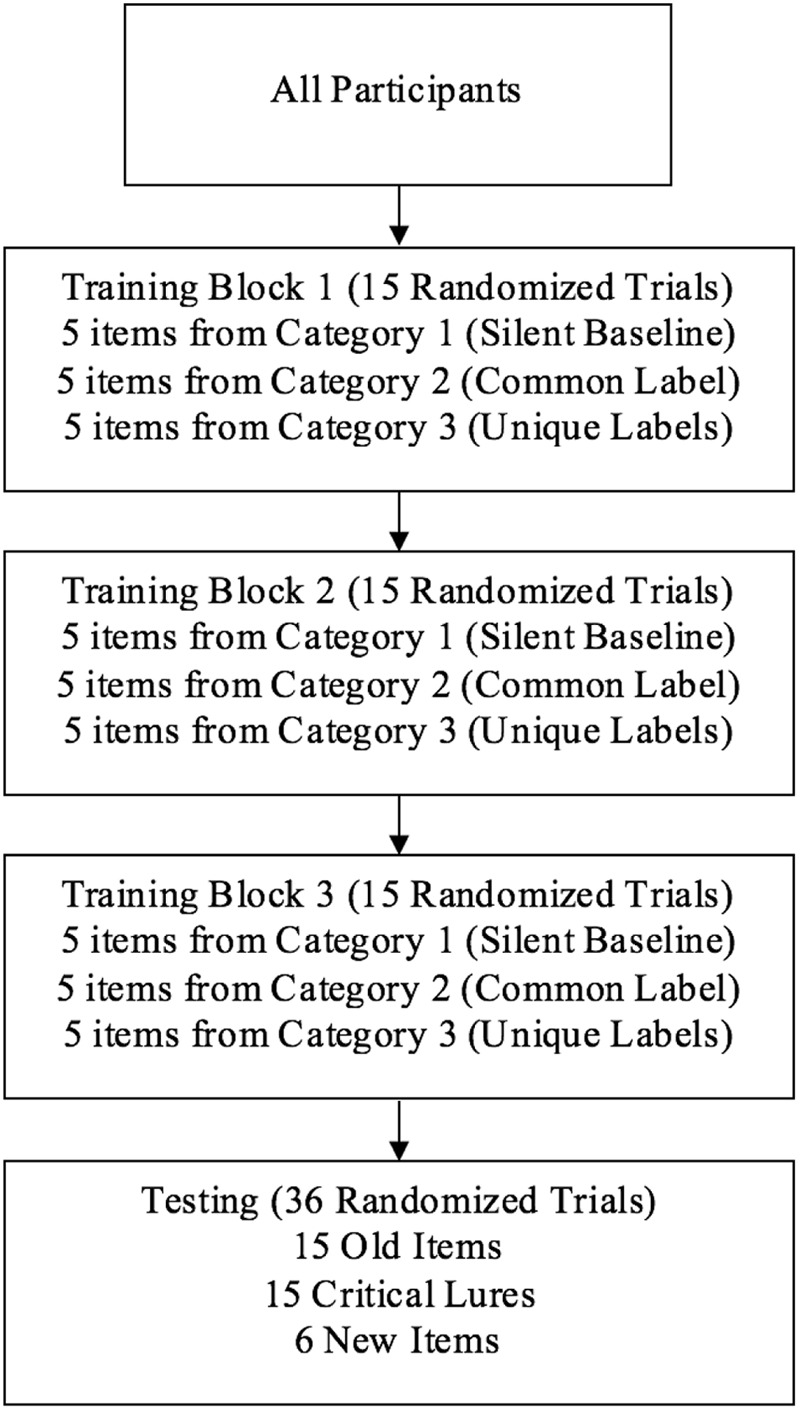
The same 15 training stimuli were presented in Blocks 1–3, and participants determined how long to view each item. Note that condition (Silent, Common, and Unique) and Category (1–3) pairings were counterbalanced across participants.

Participants were told they would see a series of creatures from another planet and learn some of the creatures’ names. They were asked to study the creatures at their own pace since they would be asked questions in the second part of the study (testing phase). They were not told about the specific recognition task, nor were they told what they would be tested on (e.g., words, creatures, word-creature pairings); however, they were informed about a test in general. Following training, participants performed an old versus new recognition task (see **Figure 3** for overview of training and testing). There were only 15 items presented throughout training, with the same 15 creatures appearing in Blocks 1–3.

Each trial began with a central drift correction target that corrected for slight eye-tracker drift during the experiment. A trained experimenter sitting at the right side of the participant initiated each trial once the participant fixated on the central cue. Stimuli were presented in a self-paced manner, in three blocks of 15 trials consisting of 5 items from each category. Participants pressed a StealthSwitch3 USB button when they were ready to move to the next stimulus. Trials within blocks were randomized, and the same 15 items were presented in each block. Each category was consistently presented in either silence, with a common label phrase, or with a unique label phrase, and category type and labeling condition were counterbalanced across participants. The auditory and visual stimuli shared the same stimulus onset. The carrier phrase and label terminated after about 1–1.5 s and the visual stimulus terminated when the participant pressed a StealthSwitch3 USB button. On average, the entire training phase (45 trials) lasted 5.38 minutes for children and 7.24 minutes for adults.

After the three training blocks, participants were presented with 36 old versus new recognition trials. There were two blocks of 18 trials, and participants were re-calibrated halfway through testing. All of the test trials were presented in silence, and only one stimulus was presented on each trial. Across the entire testing phase, participants saw five exemplars from each of the three studied categories (15 old items) and five critical lures which were associated with the three studied categories (15 critical lures). Critical lures were novel creatures with new features, but these stimuli had a category defining feature. We also presented six new “catch” stimuli. These stimuli did not have a category defining feature and they also had fewer features than the other items. For example, as can be seen in **Figure 1**, all of the old items and critical lures had four legs or two legs and two arms. New catch items did not have any legs (but some had small fins). We used six catch trials instead of five so we could have the same number of trials in testing blocks 1 and 2 (18 trials per block). Each trial began with a drift correction target as before. Participants responded old or new by pressing one StealthSwitch3 USB button for old and a different StealthSwitch3 USB button for new. Left-right button location was counterbalanced across participants. Participants were instructed to respond as fast and as accurately as possible. Response times and visual fixations were recorded during both training and test. On average, the testing phase lasted 2.54 and 2.49 minutes for children and adults, respectively.

## Results

The primary analyses focused on visual fixations during training. Based on a proposed mechanism underlying effects of labels on category learning (Waxman, 2003; Lupyan, 2012), it was hypothesized that common labels would be associated with increased attention to category relevant features and unique labels would be associated with increased attention to unique features.

### Training Data

**FIGURE 4:**
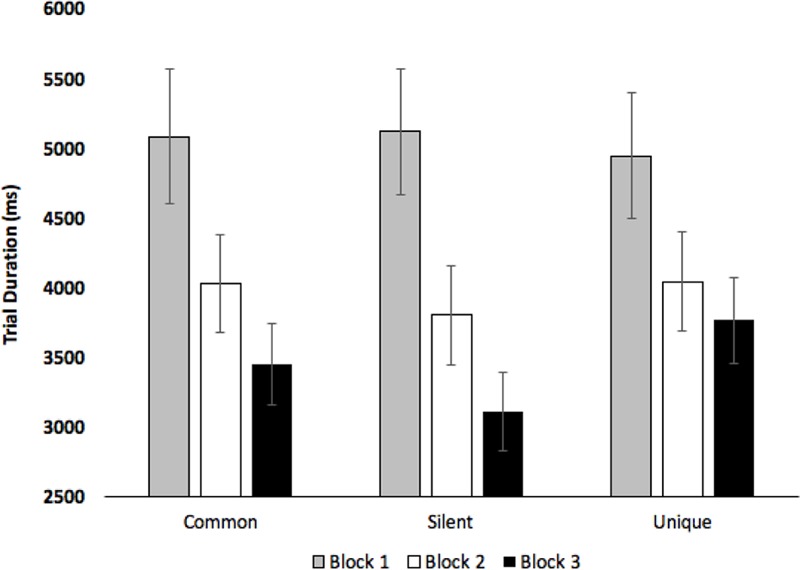
Trial duration across Condition and Block. Error bars denote Standard Errors.

**FIGURE 5:**
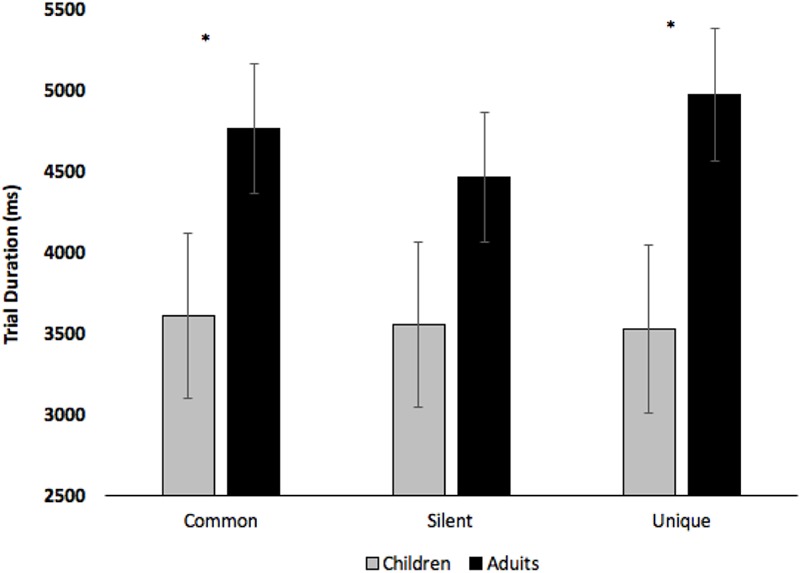
Trial duration across Condition and Age. Error bars denote Standard Errors and “^∗^” denotes that adults differed from children, *p*s < 0.053.

Initial analyses examined how long participants viewed the novel creatures - recall that participants terminated each training trial by pressing a StealthSwitch3 USB button when they were ready to view the next creature. Participants saw 15 different creatures which were repeated three times (once per block), thus, participants should spend less time viewing the images as they become more familiar. Trial duration was submitted to a 2 (Age: Children vs. Adults) × 3 (Condition: Common vs. Silent vs. Unique) × 3 (Block: Blocks 1–3) mixed-factors ANOVA, with Age manipulated between subjects. The analysis revealed an effect of Condition, *F*(2,74) = 3.20, *p* = 0.047, $\eta_{p}^{2}$ = 0.08, with participants viewing the images longer when paired with unique labels (*M* = 4248 ms, *SE* = 331) than when presented in silence (*M* = 4007 ms, *SE* = 326), pairwise *p* = 0.017. The effect of Block was also significant, *F*(2,74) = 15.34, *p* < 0.001, $\eta_{p}^{2}$ = 0.29, with viewing time decreasing across Block 1 (*M* = 5046 ms, *SE* = 447), Block 2 (*M* = 3955 ms, *SE* = 343), and Block 3 (*M* = 3439 ms, *SE* = 386), all *p*s < 0.003. The analyses revealed a marginal effect of Age, *F*(1,37) = 3.32, *p* = 0.076, $\eta_{p}^{2}$ = 0.08, with adults (*M* = 4733 ms, *SE* = 399) viewing images longer than children (*M* = 3559 ms, *SE* = 505). The analyses also revealed significant Block × Condition, *F*(4,148) = 2.85, *p* = 0.026, $\eta_{p}^{2}$ = 0.07, and Condition × Age, *F*(2,74) = 3.66, *p* = 0.030, $\eta_{p}^{2}$ = 0.09, interactions (see **Figures 4, 5**, respectively). As can be seen in **Figure 5**, effects of age were most pronounced in the common and unique label conditions.

**FIGURE 6:**
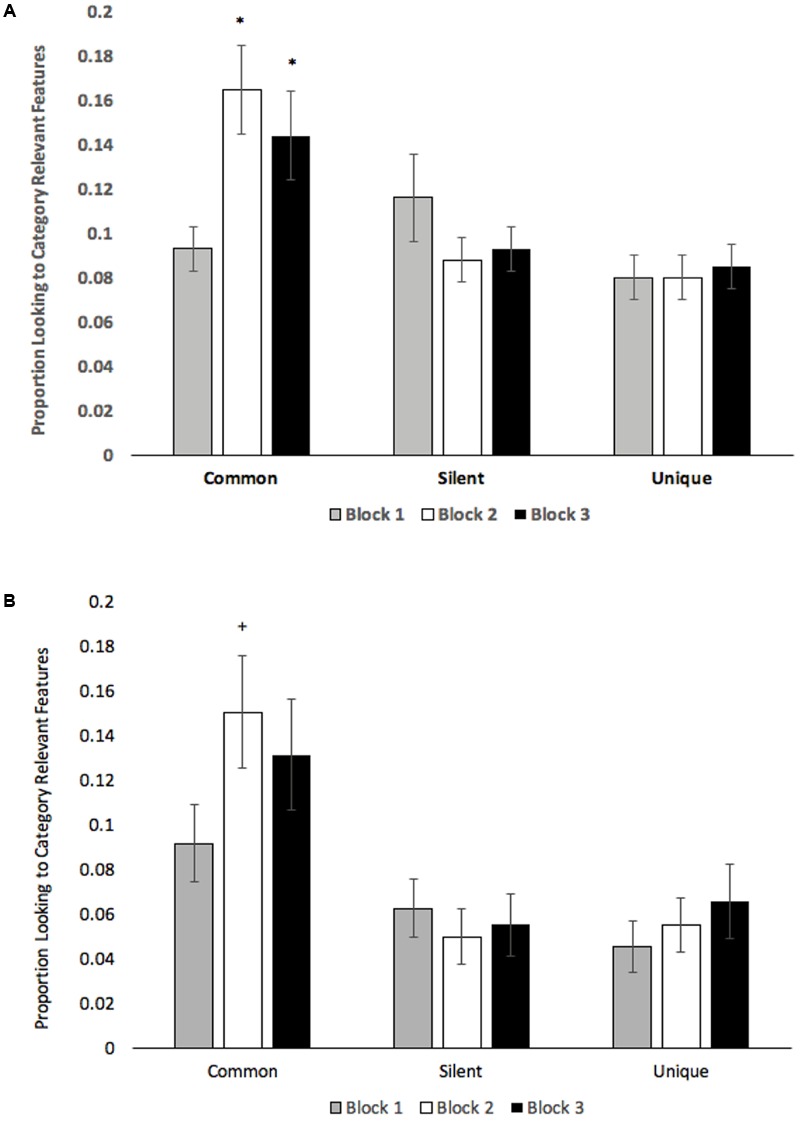
**(A)** Proportion looking to category relevant features averaged across the entire training trial and **(B)** Proportion looking to category relevant features averaged across the first 1000 ms of each training trial. Error bars denote Standard Errors. “+” denotes that the mean differed from Block 1, *p* < 0.01, “^∗^” denotes *p*s < 0.005.

To determine how labeling affects attention, we computed the proportion of looking to category relevant features on each trial. This value was calculated by summing the total fixation time at the relevant AOIs and dividing that value by the total fixation time at any location on the stimulus. Proportion of looking to category relevant features was submitted to a 2 (Age: Children vs. Adults) × 3 (Condition: Common vs. Silent vs. Unique) × 3 (Block: Blocks 1–3) mixed-factors ANOVA, with Age manipulated between subjects. The analysis revealed a main effect of Condition, *F*(2,74) = 5.36, *p* = 0.007, $\eta_{p}^{2}$ = 0.13, with participants accumulating more looking to category relevant features when presented with common labels (*M* = 0.13, *SE* = 0.01) than when images were paired with unique labels (*M* = 0.08, *SE* = 0.01), pairwise *p* = 0.004. Looking to category relevant features in common and unique conditions did not differ from the silent baseline, *p*s > 0.15. The analysis also revealed a Condition × Block interaction, *F*(4,148) = 10.20, *p* < 0.001, $\eta_{p}^{2}$ = 0.22. As can be seen in **Figure 6A**, participants only increased looking to category relevant features in the common label condition, with Blocks 2 and 3 both exceeding Block 1, *t*s (38) > 3.04, *p*s < 0.004. Given previous findings that effects of labels on attention occur prior to the presentation of the label (Althaus and Mareschal, 2014), we have also included proportion looking to category relevant features only within the first 1000 ms of each training trial (see **Figure 6B**). There was also some evidence that looking to category relevant features marginally decreased from Block 1 to Block 2 in the silent condition, *t*(38) > 2.18, *p* = 0.036, adjusted alpha = 0.016.

**FIGURE 7:**
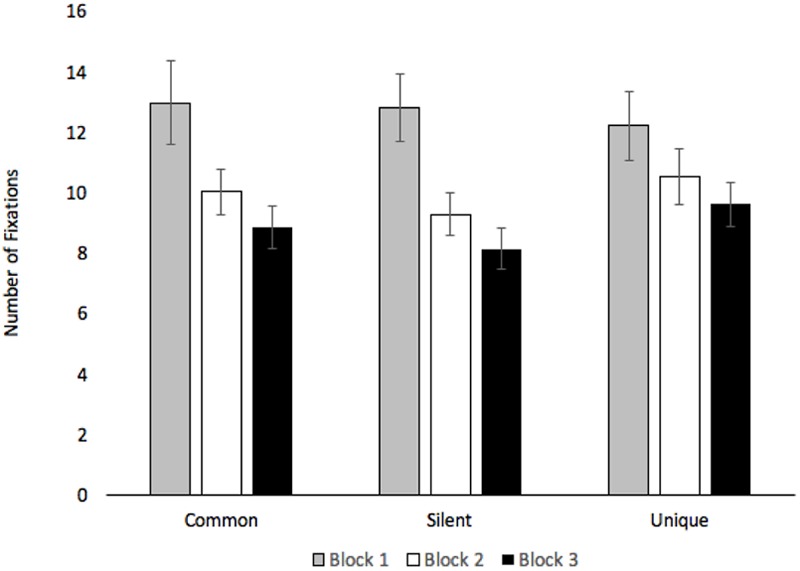
Number of fixations across Condition and Block. Error bars denote Standard Errors.

**FIGURE 8:**
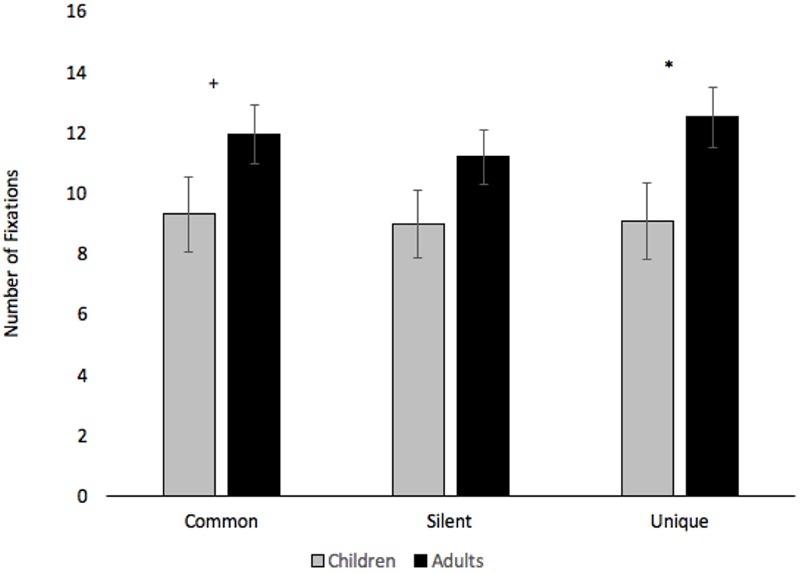
Number of fixations across Condition and Age. Error bars denote Standard Errors. “+” denotes that adults differed from children, *p* = 0.087 and “^∗^” denotes that adults differed from children, *p* < 0.05.

Number of fixations to anywhere on the stimulus were submitted to a 2 (Age: Children vs. Adults) × 3 (Condition: Common vs. Silent vs. Unique) × 3 (Block: Blocks 1–3) mixed-factors ANOVA, with Age manipulated between subjects. The findings closely parallel the trial duration analyses. The analysis revealed an effect of Condition, *F*(2,74) = 4.57, *p* = 0.013, $\eta_{p}^{2}$ = 0.11, with participants making more fixations when creatures were paired with unique labels (*M* = 10.78, *SE* = 0.79) than when presented in silence (*M* = 10.06, *SE* = 0.72), *p* = 0.005. Participants also made marginally more fixations in the common word condition (*M* = 10.60, *SE* = 0.79) than the silent baseline, *p* = 0.063. The effect of Block, *F*(2,74) = 10.92, *p* < 0.001, $\eta_{p}^{2}$ = 0.22, shows that the number of fixations decreased across Block 1 (*M* = 12.66, *SE* = 1.18), Block 2 (*M* = 9.93, *SE* = 0.76), and Block 3 (*M* = 8.85, *SE* = 0.68), all *p*s < 0.017. The analyses revealed a marginal effect of Age, *F*(1,37) = 3.33, *p* = 0.076, $\eta_{p}^{2}$ = 0.08, with adults (*M* = 11.86, *SE* = 0.95) making more fixations than children (*M* = 9.10, *SE* = 1.19). The analyses also revealed significant Block × Condition, *F*(4,148) = 3.29, *p* = 0.013, $\eta_{p}^{2}$ = 0.08, and Condition × Age, *F*(2,74) = 3.23, *p* = 0.045, $\eta_{p}^{2}$ = 0.08, interactions (see **Figures 7, 8**, respectively).

### Testing Data

On each testing trial, a single stimulus was presented and participants had to indicate if the stimulus was old (presented during training) or new (not presented during training). Averaged across participants, children responded correctly on approximately 35 out of the 36 trials (Range 32–36) and adults responded correctly on approximately 35 out of the 36 trials (Range 33–36). The proportions of correct responses were also collected separately for old items (items presented during training), critical lures (new items that had the category relevant feature) and new catch items. Only participants who correctly identified at least four of the six catch trials as new were included in the following analyses. Data from four children were excluded from testing analyses: one child was excluded because s/he missed all of the catch trials (response bias), and three children were excluded due to experimental error (corrupt testing files). Of the remaining participants, we calculated discrimination accuracies by calculating the proportion of hits on old items and subtracting the proportion of false alarms on critical lures (i.e., proportion of hits – proportion of false alarms). Accuracies were submitted to a 2 (Age: Children vs. Adults) × 3 (Condition: Common vs. Silent vs. Unique) mixed-factors ANOVA. All accuracies were greater than 0.94, and the analyses revealed no significant effects.

**FIGURE 9:**
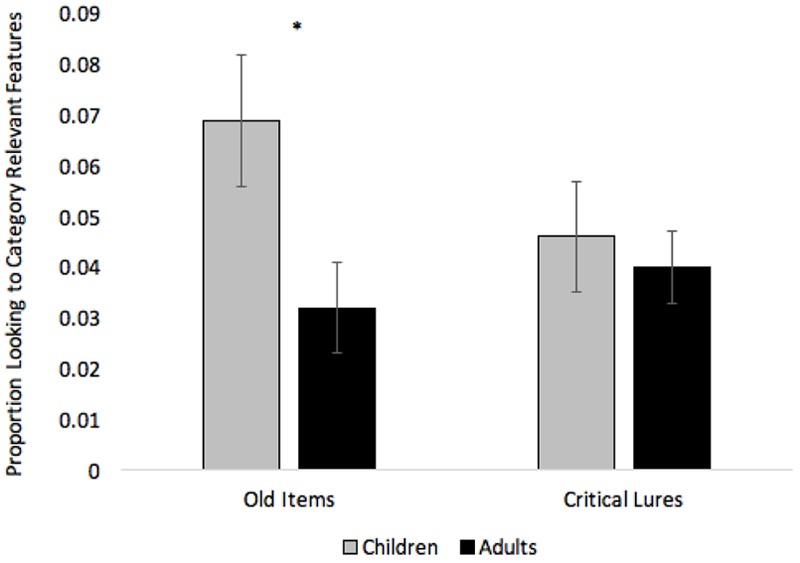
Proportion looking to category relevant features at test across Age and Trial Type. Error bars denote Standard Errors and “^∗^” denotes that adults differed from children, *p* < 0.01.

We also submitted response times, number of fixations, mean fixation durations, and proportion looking to relevant AOIs to separate 2 (Children vs. Adults) × 3 (Condition: Common vs. Silent vs. Unique) × 2 (Trial Type: Old item vs. Critical Lure) mixed-factors ANOVAs. The response time and number of fixation analyses only revealed effects of Age, *F*s(1,33) > 9.60, *ps* < 0.004, $\eta_{p}^{2}$ > 0.24, with adults (*M* = 950, *SE* = 78) responding faster than children (*M* = 1430 ms, *SE* = 115) and adults (*M* = 2.58, *SE* = 0.23) making fewer fixations than children (*M* = 3.83, *SE* = 0.34). The fixation duration analysis only revealed an effect of Trial Type, *F*(1,33) = 19.17, *p* < 0.001, $\eta_{p}^{2}$ = 0.37, with participants making longer fixations to old items (*M* = 336 ms, *SE* = 18) than critical lures (*M* = 288, *SE* = 11). The looking to category relevant features analysis revealed an effect of Condition, *F*(2,66) = 5.58, *p* = 0.006, $\eta_{p}^{2}$ = 0.15, with participants looking more to category relevant features on common items (*M* = 0.07, *SE* = 0.01) compared to unique items (*M* = 0.02, *SE* = 0.01), *t*(34) = 2.52, *p* = 0.016. Thus, some of the effects of labeling during training carried over and affected looking while participants were making same-different responses at test. The analysis also revealed a marginal effect of Age, *F*(1,33) = 3.42, *p* = 0.073, $\eta_{p}^{2}$ = 0.09, and an Age × Trial Type interaction, *F*(1,33) = 3.56, *p* = 0.067, $\eta_{p}^{2}$ = 0.10. Compared to adults, children looked marginally more to category relevant features while making old-new judgments at test, and the interaction suggests that this developmental effect was more pronounced when presented with old items than critical lures (see **Figure 9**).

## Discussion

The present study investigated if simply hearing linguistic labels, particularly common and unique labels, direct attention to category relevant features in children and young adults. Previous research examining the effects of labels on cognitive tasks show that both common and unique labels can affect the *outcome* of learning, and it is often assumed that these effects stem from labels directing attention to category relevant features (cf. Waxman, 2003). However, recent eye tracking studies in infants and young children directly testing this hypothesis have yielded mixed results. More specifically, some research has identified that common labels direct attention to commonalities (Althaus and Mareschal, 2014; Althaus and Plunkett, 2015a,b; Althaus and Westermann, 2016), whereas other studies have demonstrated that infants and children show a preference to look at the changing, unique features, with no evidence that labels direct attention to commonalities (Best et al., 2010, 2011). Moreover, while it is well-established that labeling affects category learning in adults (Lupyan et al., 2007) and adults can optimize their attention in a categorization task where they make category judgments with feedback (Blair et al., 2009), to the best of our knowledge, no research has demonstrated that simply hearing labels associated with objects directs adults’ attention to category relevant features.

To address these issues, the present study employed a novel procedure to examine how labels affect attention in children and adults. Most categorization studies with children and adults typically make it explicit that participants will be presented with different exemplars from various categories, require participants to make category judgments about each item, and then corrective feedback is often provided (see Rehder and Hoffman, 2005; Blair et al., 2009, for examples). However, we were interested if labeling alone can result in attention optimization; thus, we used a paradigm that is more similar to infant familiarization studies. Participants were not informed about the underlying categories, nor were they expected to make category judgments. Rather, we simultaneously “familiarized” children and adults to three different kinds of creatures and participants heard some of the creatures’ names. To examine if labels directed attention to category relevant features, we recorded participants’ visual fixations while they passively viewed the creatures.

Based on a proposed mechanism underlying effects of labels on category learning (Waxman, 2003; Lupyan, 2012), it was hypothesized that hearing the same label associated with different exemplars would direct attention to category relevant (common) features. Support for this finding was identified in both children and young adults, in which participants accumulated more overall looking to common features when images were paired with common labels, and common labels appeared to direct attention to category relevant features over time as evidenced by increased looking to common features in Blocks 2 and 3 (**Figure 6**). Participants also viewed images for longer durations and made more fixations when creatures were paired with unique labels.

The present study also sought to examine recognition of individual items at test to determine if effects of labels carry over to non-categorization tasks. Previous research with 4-year-olds found that discriminability of images paired with labels during training decreased on a subsequent testing phase, even though no labels were provided at test (Sloutsky et al., 2005). In contrast, effects of labeling during training did not attenuate subsequent discrimination in adults. If participants were primarily focusing on category relevant features in the training phase of the present study, then false alarms to critical lures should have increased due to falsely recognizing critical lures as old because they shared the common feature. The present study did not find a decrease in recognition accuracy in the common label condition; however, children in the present study were older than the children in Sloutsky et al. (2005), and there were numerous methodological differences complicating direct comparisons (use of dense versus sparse categories, testing recognition memory versus simple discrimination, etc.). That said, there was some evidence that participants showed different patterns of looking on a recognition task with more looking to common features when the same label was associated with each member of the category during training. This suggests that some effects of labels may be more general in nature and have subsequent effects on a variety of cognitive tasks.

We found support for label induced attention optimization for sparse categories in 8-year-olds and adults; however, it is unclear if this effect will be found in younger populations. One possible reason is because learning of rule-defined or sparse categories is especially difficult for young children (Kloos and Sloutsky, 2008). This likely stems from young children not having the attentional control to focus on a small subset of category relevant features while simultaneously ignoring many irrelevant features. Another possible reason why label induced attention optimization to sparse categories seems unlikely in infancy stems from auditory dominance research, which shows that words and sounds can sometimes disrupt visual processing in development (Lewkowicz, 1988a,b; Sloutsky and Napolitano, 2003; Robinson and Sloutsky, 2004, 2007b; Sloutsky and Robinson, 2008; Nava and Pavani, 2013), with infants sometimes being more likely to learn visual categories when no auditory information is provided (Robinson and Sloutsky, 2007a). However, at some point in development, effects of labeling on visual attention should interact with category sparsity. For example, when examining the outcome of learning, labels have little to no facilitative effects on basic-level categories which can be learned without supervision, while at the same time, they appear to have greater effects on global categorization (Waxman and Markow, 1995; Fulkerson and Haaf, 2003). Thus, future research will need to systematically manipulate category sparsity to examine if these facilitative effects of labels on categorization stem from increased attention to category relevant features (we revisit this below).

There are several limitations of the present study, and we have also provided suggestions for future research. First, future research will need to systematically manipulate instructions, task demands, and feedback to see how each of these components affects attention optimization. For example, the present study found label induced attention optimization when participants were provided with ambiguous instructions and were not informed about underlying categories. Would attention optimization effects increase if participants were explicitly told about the underlying category structure and instructed that they would be asked about the types of creatures at test?

Second, the present study also did not require participants to make category judgments, whereas many categorization studies require participants to classify each object and feedback is provided. This is important because most models of categorization often assume that attention optimization occurs as a means to reduce error (see Rehder and Hoffman, 2005; Blair et al., 2009; for related discussions). Participants in the present study did not classify each object and no feedback was provided; thus, there was no error to reduce. This suggests that attention optimization is either not contingent on error reduction or participants were using the labels as supervisory signals (e.g., predicting category membership and then using label as feedback). Future research will need to use a similar paradigm and test categorization to examine if labels are serving as: (a) supervisory signals, (b) features which increase overall similarity of compared entities (Sloutsky and Lo, 1999; Sloutsky et al., 2001; Sloutsky and Fisher, 2004), or (c) highly salient auditory features that overshadow encoding of unique features (Sloutsky et al., 2005). While all three could facilitate categorization, only supervisory signals should be linked to increased attention to category relevant features during training. Training data are consistent with the supervisory signal account, but future research will also need to show that effects of labels also change the outcome of learning.

Third, in the present study, we used perceptually rich stimuli, which contained many different features/dimensions, and stimuli were also presented at different orientations. While stimuli were diverse and engaging, one limitation of our choice of stimuli was that we had less control over the relative size and number of the AOIs. For example, stimuli in Category A were defined by two three-pronged features which were located around the shoulders; however, depending on stimulus orientation, one or two of these features were visible. Stimuli in Category C were defined by suction cup feet. Creatures with four legs had four AOIs, whereas creatures with two legs and two arms only had two AOIs. In contrast, there was always one relevant AOI in Category B, which was always visible (dorsal fin). This variability resulted in different amounts of looking to the relevant AOIs; however, this did not simply map onto larger or more AOIs resulting in more overall looking. Rather, collapsed across labeling conditions, participants accumulated more looking to the three-prong feature (0.15) than the suction cup feet (0.11) and dorsal fin (0.05). However, it is important to note that Categories (A, B, or C) and Labeling (Common, Unique, and No label) pairings were counterbalanced across participants; thus, variability in AOIs cannot account for the reported findings that participants increased looking to category relevant features in the common label condition. Furthermore, across all three possible category-label pairings, participants who heard the same label associated with different category members increased looking to category relevant features in Blocks 2 and 3, relative to Block 1. This suggests that the effect is robust and not tied to a specific feature.

Finally, and related to a previous point, the present study did not assess participants knowledge of the underlying categories, nor did we ask participants to state which features were important for determining category membership. Data collection addressing this issue is underway and is important for several reasons. First, without this data, it is unclear if shifts in attentional weights also correspond with explicit learning of the categories. Thus, it is possible that attention was shifted to category relevant features in an implicit, bottom-up manner, with no explicit knowledge of the underlying categories. Second, by assessing categorization at test, it will be possible to look at individual patterns of learning. For example, do patterns of visual fixations differ between learners and non-learners? Do children and adult participants both optimize attention and learn the categories or is it possible to learn the categories without having the attentional control to optimize attention?

In summary, the effects of labeling on category learning are well documented; however, research examining the underlying mechanisms are poorly understood. We used a novel paradigm to examine fixations to category relevant features across training. Common labels directed attention to category relevant features in both 8-year-olds and adults, which is consistent with a proposed mechanism of effects of labels on categorization (Waxman, 2003; Lupyan, 2012). The presence of unique labels also increased exploration of stimuli, with participants making more fixations and having longer trial durations when each member of the category was associated with a different label. Finally, there was some evidence that these changes in attentional weights persisted beyond the labeling episode, which may have lasting effects on how the labeled objects are perceived and categorized.

## Ethics Statement

This study was carried out in accordance with the recommendations of Behavioral and Social Science Institutional Review Board at The Ohio State University, with written informed consent from all subjects. All subjects gave written informed consent in accordance with the Declaration of Helsinki. The protocol (2014B0022) was approved by the Behavioral and Social Science Institutional Review Board at The Ohio State University.

## Author Contributions

All authors listed have made a substantial, direct and intellectual contribution to the work, and approved it for publication.

## Conflict of Interest Statement

The authors declare that the research was conducted in the absence of any commercial or financial relationships that could be construed as a potential conflict of interest.
